# Association of albumin levels with the risk of intracranial atherosclerosis

**DOI:** 10.1186/s12883-023-03234-2

**Published:** 2023-05-20

**Authors:** Xiaoyu Lin, Fangfang Ke, Maohua Chen

**Affiliations:** Department of Neurosurgery, Wenzhou Central Hospital Affiliated Dingli Clinical Institute of Wenzhou Medical University, Wenzhou, China

**Keywords:** Serum albumin, Intracranial artery stenosis, Atherosclerosis, Risk factors

## Abstract

**Objective:**

Intracranial artery stenosis from atherosclerosis is one of the etiologies of ischemic stroke. There is a correlation between serum albumin level and atherosclerosis. We aimed to investigate whether serum albumin level is related to intracranial atherosclerosis and its significance.

**Methods:**

A retrospective analysis of 150 individuals who underwent cervical cerebral angiography after admission, including clinical data, imaging data, and laboratory data. Since atherosclerosis cannot be used as a good quantitative indicator, we choose the degree of arterial stenosis to reflect atherosclerosis. SPSS 24 software was used for data analysis, and P < .05 was considered statistically significant.

**Results:**

Univariate analysis showed that age, diabetes, and serum albumin level were risk factors for intracranial atherosclerosis (P < .05). Multivariate analysis showed that diabetes and serum albumin levels were independent risk factors for intracranial atherosclerosis (P< 0.05). The average serum albumin level in the non-severe group was 39.80 g/L, and the average serum albumin level in the severe group was 37.60 g/L. The area under the ROC curve of serum albumin was 0.667 (95%CI 0.576–0.758, P = .001), the cutoff value was 0.332176, the sensitivity was 75.9%, and the specificity was 57.3%.

**Conclusion:**

Serum albumin level is an independent risk factor for intracranial atherosclerosis, and provides a new direction for clinical prevention and treatment.

## Introduction

Ischemic stroke is one of the most common cerebrovascular conditions. In the wake of rapid social development, people’s lifestyles and eating habits have gradually changed, including lack of exercise, increased work pressure, high-fat and high-salt diet, etc. The incidence of ischemic stroke has increased globally each year [1]. In China, approximately 7 million patients suffer from stroke, of which > 70% suffer from ischemic stroke. A recent study found that from 2013 to 2019, the weighted prevalence of stroke in China increased year by year, and it was 2.28% (95% CI: 2.28%-2.28%) in 2013, 2.34% (2.34-2.35%) in 2014, 2.43% (2.43%-2.43%) in 2015, 2.48% (2.48%−2.48%) in 2016, 2.52% (2.52%−2.52%) in 2017, 2.55% (2.55%−2.55%) in 2018%, and 2.58% (2.58%−2.58%) in 2019 (p < .001) [2]. The recurrence rate of ischemic stroke is about 8%, and the disability rate is as high as 17%, which is the main cause of neurological dysfunction. Ischemic stroke not only affects the patient’s self-care ability, but also brings a heavy burden to the family and society [3]. Therefore, early detection and prevention of ischemic stroke has important practical significance. Intracranial artery stenosis from atherosclerosis is on the etiologies of ischemic stroke and hypertension, hyperlipidemia and hyperglycemia are considered as risk factors to intracranial atherosclerosis [4]. Recent studies have found that serum albumin levels are associated with atherosclerosis, with lower albumin levels exacerbating atherosclerosis and increasing the risk of hospitalization and death, independent of pre-existing disease [5]. Therefore, we hypothesized that serum albumin levels may be associated with intracranial atherosclerosis, but atherosclerosis was not a good quantifiable indicator. In view of the fact that atherosclerosis can cause arterial stenosis, the degree of atherosclerosis can be reflected by the degree of arterial stenosis, so as to study the relationship between serum albumin level and intracranial atherosclerosis, in order to prevent, delay or treat intracranial atherosclerosis or stenosis. The purpose of this study was to investigate whether serum albumin levels are related to intracranial atherosclerosis and its significance.

## Materials and methods

### Clinical information

**Fig. 1:**
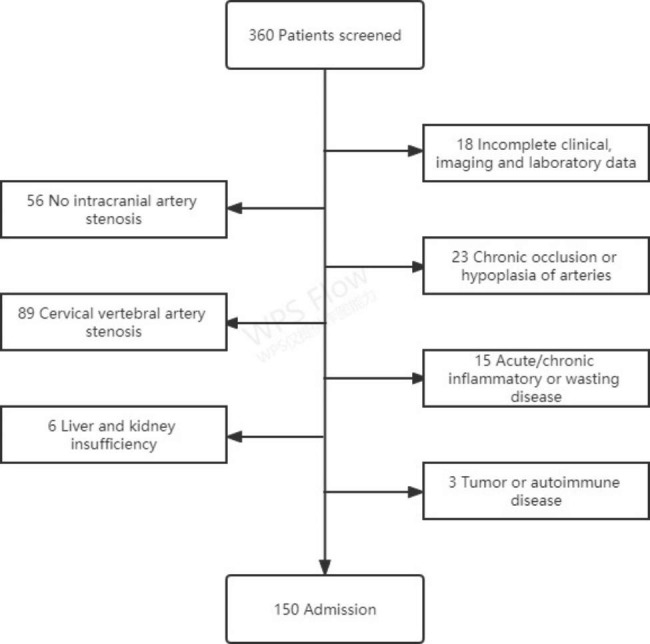
Flow chart of patients included in the study. From June 2020 to March 2021, a total of 360 patients underwent cervical cerebral angiography were screened. During the screening, those with incomplete clinical, imaging and laboratory data, no intracranial artery stenosis, chronic total occlusion or hypoplasia of arteries, cervical vertebral artery stenosis, acute/chronic inflammatory or wasting diseases, liver and kidney insufficiency, tumor or autoimmune diseases will be excluded, 150 cases were finally included

A retrospective analysis was performed on patients who underwent cervical cerebral angiography in the Department of Neurosurgery of Wenzhou Central Hospital from June 2020 to March 2021. Inclusion criteria: (1) Complete cervical cerebral angiography after admission, including stroke patients; aneurysm patients, including ruptured or unruptured patients with intracranial vascular stenosis found in DSA examination; asymptomatic examination patients, etc.; (2) Clinical, imaging and laboratory related data are complete. Exclusion criteria: (1) Patients with chronic total arterial occlusion or hypoplasia; (2) Patients with carotid artery or vertebral artery stenosis at the same time; (3) Acute/chronic inflammatory or wasting disease at the time of admission, including any focal or systemic disease Infection; (4) Severe liver and kidney insufficiency; (5) Past or current tumor or autoimmune disease. A total of 360 cases were screened in this study, including 218 males, accounting for 60.56%; 142 females, accounting for 39.44%, aged (62.56 ± 12.01) years; 150 cases were included, including 93 males and 57 females; age (62.35 ± 12.01) years old. 210 cases were excluded. (Fig. 1)

### Image data

All the included cases underwent cervical cerebral angiography after admission, and the degree of intracranial arterial stenosis was interpreted by two physicians with rich clinical experience through WASID method. At present, the Chinese guidelines for carotid stenosis management released in 2017 suggested that for patients with carotid stenosis, the degree of stenosis ≥ 50% should be re-examined and the development of carotid stenosis should be monitored. Therefore, referring to this recommendation, we divided the degree of intracranial artery stenosis into non-severe stenosis (stenosis < 50%) and severe stenosis (stenosis ≥ 50%), and the patients were divided into two groups: non-severe group (n = 96) and severe group (n = 54).

### Laboratory data

After admission, 10 ml of venous blood was collected after 10 h of fasting, and the red blood cell, white blood cell, platelet counts and basic biochemical clinical data were analyzed with an automatic hematology analyzer.

### Statistical analysis

SPSS 24 software was used for data analysis, measurement data were expressed as “X̅ ± S”, and Student’s t-test was used for comparison. Enumeration data were expressed as percentages and compared using the X^2^ test. Multiple factors were analyzed by binary Logistics regression, and P < .05 was considered statistically significant. The ROC curve of serum albumin was established, and the area under the curve and the 95% confidence interval were calculated.

## Results

### Univariate analysis

Univariate analysis showed that age, diabetes, and serum albumin level were risk factors for intracranial atherosclerosis (P < .05). Gender, smoking, drinking, hypertension, coronary heart disease, red blood cell count, white blood cell count, platelet count, serum globulin level, A/G, total cholesterol level, triglyceride level, homocysteine level were not significantly correlated with intracranial atherosclerosis (P > .05). (Table 1)

**Table 1:** Univariate analysis of the degree of intracranial atherosclerosis

Factor	Non-severe group(n = 96)	Severe group(n = 54)	P
Sex, male(%)	58(60.42)	35(64.81)	0.595
Smoking(%)	17(17.71)	17(31.48)	0.054
Drinking(%)	16(16.67)	10(18.52)	0.774
Hypertension(%)	58(60.42)	40(74.07)	0.093
Diabetes(%)	20(20.83)	23(42.59)	0.005
CHD(%)	7(7.29)	4(7.41)	0.979
Age, years	60.57 ± 13.53	65.50 ± 8.27	0.031
Red blood cell count	4.45 ± 0.47	4.37 ± 0.42	0.375
White blood cell count	6.96 ± 2.31	6.88 ± 1.86	0.820
Platelet count	219.77 ± 72.69	208.11 ± 53.76	0.514
Serum albumin level	39.84 ± 3.74	37.80 ± 3.72	0.001
Serum globulin level	28.36 ± 3.85	27.74 ± 4.42	0.232
A/G	1.43 ± 0.21	1.38 ± 0.18	0.189
Total cholesterol level	4.83 ± 1.24	4.58 ± 1.31	0.206
Triglyceride level	1.77 ± 1.32	1.69 ± 0.85	0.527
Homocysteine level	12.88 ± 5.90	13.13 ± 5.31	0.769

**Fig. 2:**
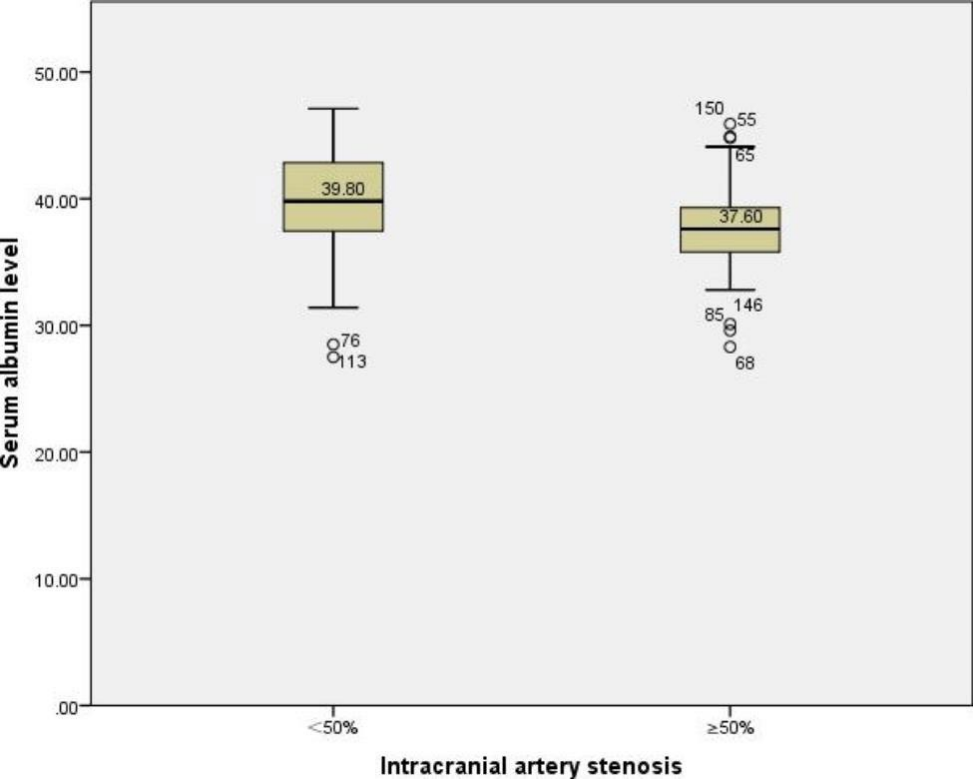
Relationship between degree of intracranial arterial stenosis and serum albumin level. After excluding individual abnormal data, the albumin level in the intracranial artery stenosis < 50% group was higher than that in the ≥ 50% group

The serum albumin levels of the non-severe group and the severe group were drawn as box plots. After excluding individual abnormal data, it was suggested that the average serum albumin level in the non-severe group was 39.80 g/L, and the average serum albumin level in the severe group was 37.60 g/L. (Fig. 2)

### Multi-factor analysis

Taking age, diabetes and serum albumin levels as independent variables, and intracranial atherosclerosis as dependent variables, binary Logistics analysis showed that diabetes and serum albumin levels were independent risk factors for intracranial atherosclerosis (P < .05). (Table 2)

**Table 2:** Multivariate analysis of the degree of intracranial atherosclerosis

Factor	B	S.E.	P	OR	95%CI	
					Lower	Upper
Age	0.027	0.018	0.126	1.027	0.992	1.063
Diabetes	0.912	0.386	0.018	2.490	1.169	5.303
Serum albumin level	-0.119	0.051	0.020	0.888	0.803	0.982

### Predictive value of serum albumin level in intracranial atherosclerosis

**Fig. 3:**
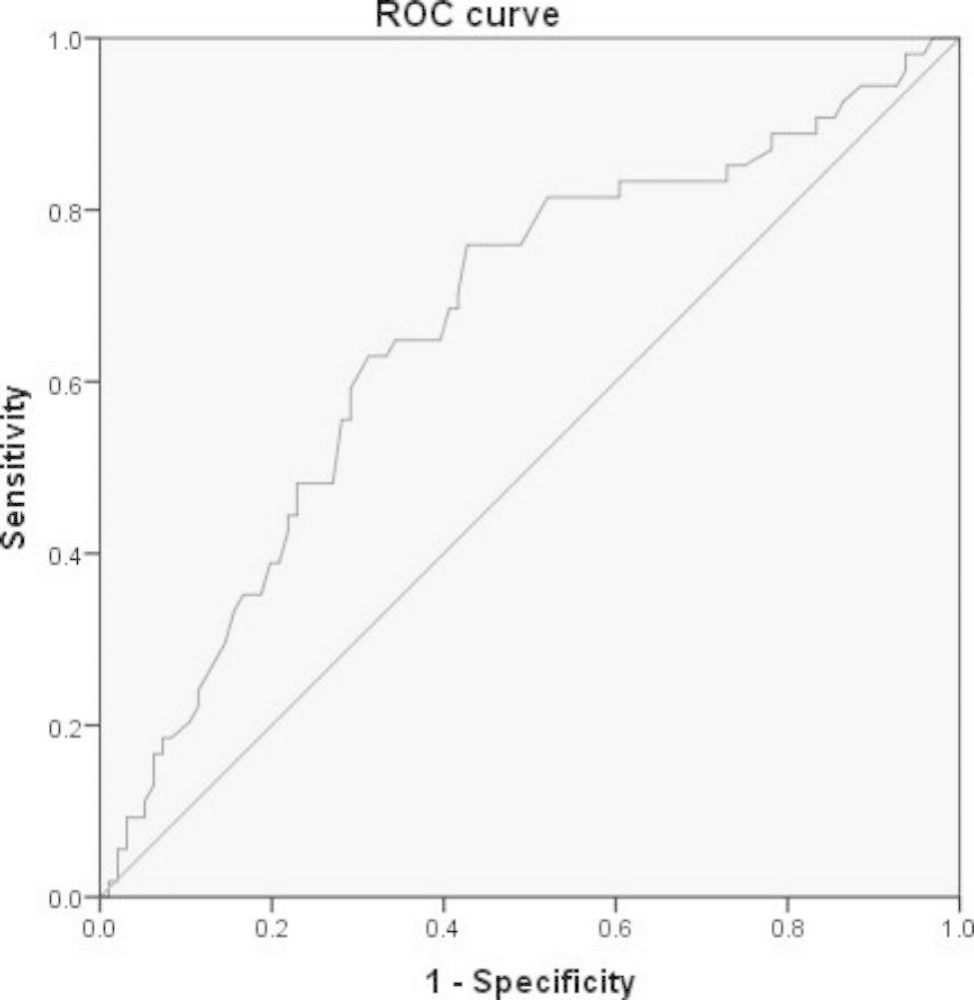
The predictive value of serum albumin level on intracranial atherosclerosis, the area under the ROC curve was 0.667, the sensitivity of serum albumin level was 75.9%, and the specificity was 57.3%

The area under the ROC curve was 0.667 (95%CI 0.576–0.758, P = .001), the cutoff value of serum albumin level was 0.332176, the sensitivity was 75.9%, and the specificity was 57.3%. (Fig. 3)

## Discussion

This study found that serum albumin level was an independent risk factor for intracranial atherosclerosis. The serum albumin level of patients with severe intracranial atherosclerosis was significantly lower than those in mild patients, and the difference was statistically significant (P < .05).

Arterial stenosis is the result of the progression of atherosclerosis. Atherosclerosis is a chronic non-regressive low-grade aseptic inflammation. The inflammatory response can reduce the stability of plaque in the artery. The more severe the reaction, the worse the stability of the plaque. Studies have shown that IL-1 is involved in the progression of atherosclerosis, and IL-1 deficiency can reduce atherosclerotic plaque and inhibit the development of atherosclerosis. Elevated levels of IL-10 and IL-17 can inhibit the progression of atherosclerosis and significantly reduce atherosclerotic plaque, while deficiency can accelerate atherosclerotic plaque formation and atherosclerotic plaque instability [6].

Albumin is the most important protein in human plasma, which maintains the body’s nutrition and osmotic pressure, accounting for about 50% of the total plasma protein. Serum albumin has many physiological roles, including being the main circulating antioxidant in the body [7]. Serum albumin is rich in thiol groups, accounting for more than 80% of the total thiols in plasma clearance of reactive oxygen and nitrogen species. Studies have shown that serum albumin also has anticoagulant and antiplatelet aggregation activities [8]. Another study showed that serum albumin is able to bind to a number of ligands, including bilirubin, long-chain fatty acids, homocysteine, copper and iron, preventing them from causing the oxidative reactions that are already involved in atherosclerosis known risk factors [9]. Serum albumin also has the ability to bind lipopolysaccharides, other bacterial proteins and other inflammatory mediators [10].

Hypoalbuminemia is mostly due to decreased hepatic synthesis, increased catabolism, increased vascular permeability, and renal and intestinal loss [11]. The relative contributions of these different mechanisms have not been investigated in patients with cerebrovascular disease. However, malnutrition and inflammation are thought to play a major role in the development of hypoalbuminemia. Studies have shown that inflammation can inhibit the synthesis of albumin, resulting in lower serum albumin levels [12].

A study of the association between low serum albumin levels and poor functional outcome and mortality in patients with AIS or TIA included 13,618 patients. During the 3-month follow-up period, patients in the < 35 g/L group had an increased risk of adverse functional outcomes and mortality compared with albumin levels between 40 g/L and 44.9 g/L (adjusted OR 1.37 (95% CI 1.12 to 1.67); adjusted HR 2.13 (95% CI 1.41 to 3.23). In the meta-analysis, for every 1 g/L reduction in albumin levels, 3 studies showed an OR of 1.03 for poor functional outcome (95% CI 1.02 to 1.05), the HR for mortality was 1.07 (95% CI 1.03 to 1.11) for five studies [13].

Therefore, this study found that hypoalbuminemia is an independent risk factor for intracranial atherosclerosis, and albumin, as the main antioxidant in the human body. It can lead to the progression of atherosclerosis, which in turn develops into arterial stenosis, causing AIS or TIA. On the other hand, the inflammatory response that exists during the progression of atherosclerosis can inhibit the synthesis of albumin, resulting in a decrease in the level of albumin and further aggravation of atherosclerosis. Therefore, how to break this vicious circle highlights its significance.

### Clinical significance

Serum albumin concentration is a simple and inexpensive routine laboratory test that provides relevant risk information for patients with certain cerebrovascular diseases. It is reasonable to speculate that when serum albumin levels are low, exogenous albumin supplementation or enhanced nutrient intake to increase albumin levels can inhibit the progression of atherosclerosis, while the inhibition of inflammatory response also reduces albumin consumption, thus forming a virtuous circle. The above speculation still needs to be verified by further prospective studies.

### Limit

In this study, serum albumin levels and intracranial artery conditions were obtained at the time of admission, and the time-varying relationship between serum albumin levels and intracranial arteries in the same patients for several months to years still needs to be obtained later to verify the study result.

This study is a single-center study with a small sample size, so the results of the study still need to be confirmed by multi-center and large-sample studies.

## Data Availability

The datasets used and/or analysed during the current study available from the corresponding author on reasonable request.
